# A Prospective Cohort Study of Changes in Access to Contraceptive Care and Use Two Years after Iowa Medicaid Coverage Restrictions at Abortion-Providing Facilities Went into Effect

**DOI:** 10.1007/s11113-022-09740-4

**Published:** 2022-09-03

**Authors:** Megan L. Kavanaugh, Mia Zolna, Emma Pliskin, Katrina MacFarlane

**Affiliations:** 1 Research Division, Guttmacher Institute, 125 Maiden Lane, 7thFloor, New York, NY 10038 USA; 2 Formerly of the Guttmacher Institute, New York, USA

**Keywords:** Contraception, Health care access, Medicaid, Iowa, Policy

## Abstract

Inequities in access to contraception based on ability to pay can interfere with individuals’ reproductive autonomy. This study examines the impact of a 2017 state-level policy in Iowa restricting Medicaid coverage at abortion-providing health care centers on patients’ access to contraceptive care and subsequent contraceptive use. We draw on a unique panel dataset of individuals who originally sought care at a publicly supported family planning site in Iowa in 2018–2019 and then participated in subsequent follow-up surveys every 6 months for 2 years to examine an effect of access to care on contraceptive use. Among our final analytic sample of 368 individuals, our findings indicate that receipt of recent contraceptive care decreased over the study period; this coincided with patients shifting away from getting contraceptive care at sites potentially impacted by the 2017 Iowa Medicaid policy restriction while those getting this care at non-impacted sites remained relatively steady over the study period. At the same time, nonuse of contraception increased while use of a contraceptive method that carries cost, use of a provider-involved method, and satisfaction with one’s method decreased. We find that, after controlling for patient characteristics, those who shifted toward receiving contraceptive care experienced increases in these three contraceptive outcomes. We interpret this as preliminary descriptive evidence demonstrating an impact of disruptions in access to contraceptive care on contraceptive outcomes. Supportive payment and funding strategies for contraception, rather than policies that impede or restrict access, are needed to enable people to realize full reproductive autonomy.

## Introduction

Contraception provides wide-ranging health, social, and economic benefits (Kavanaugh & Anderson, 2013; Sonfield et al., 2013) and is a key tool for many individuals’ realization of reproductive autonomy. Of the almost 73 million women of reproductive age in the United States (US) in 2018, 46 million were both sexually active and not seeking pregnancy (“Contraceptive Use in the United States by Method” 2021), a group for whom contraception could help achieve their reproductive goals. However, inequities in access to contraception based on ability to pay can interfere with individuals’ ability to take advantage of these benefits.

In 2017, Iowa legislators implemented a state-level Medicaid family planning program that removed funding eligibility from health care sites that provided abortion care or referrals and, as such, removed the ability of patients enrolled in this state family planning program to access subsidized sexual and reproductive health care (SRH) at these sites. Given the critical role that contraception plays in an individual’s ability to realize reproductive autonomy, we hypothesized that the 2017 changes to Medicaid coverage in Iowa would lead to disruptions in people’s access to SRH care and, subsequently, decreases in contraceptive use. Most existing evidence on the relationship between access to care and contraceptive use is cross-sectional and therefore correlational. In contrast, we exploit a unique panel dataset of individuals who originally sought care at a publicly supported family planning site in Iowa in 2018 and then participated in subsequent follow-up surveys every 6 months for 2 years to examine an effect of access to care on contraceptive use.

In this paper, we first review evidence on cost as a barrier to contraceptive access. We provide examples of other state-level policies that have impacted contraceptive access, and we review the specific Iowa policy implemented in 2017 that restricted patients’ ability to use their Medicaid coverage at specific family planning care sites. We describe our data collection protocols to recruit patients at Iowa health care centers that received public funding to provide family planning care into a longitudinal survey panel covering 2 years following the policy’s implementation. We highlight several ways in which we operationalize both the key mediator construct of access to contraception and the outcome of contraceptive use in our analysis. Among this sample of Iowa family planning patients, our findings indicate that receipt of recent contraceptive care decreased over the study period; this coincided with patients shifting away from getting contraceptive care at sites that may have been impacted by the 2017 Iowa Medicaid policy restriction while those getting this care at non-impacted sites remained relatively steady over the study period. At the same time, nonuse of contraception increased while use of a contraceptive method that carries cost, use of a provider-involved method, and satisfaction with one’s method decreased. We find that, after controlling for patient characteristics, those who shifted toward receiving contraceptive care—either at a site potentially impacted by the 2017 Iowa Medicaid policy restriction or at a non-impacted site—experienced shifts toward using contraceptive methods that carry cost, provider-involved methods and satisfaction with the method used. We interpret this as preliminary descriptive evidence demonstrating an impact of disruptions in access to contraceptive care on contraceptive outcomes.

## Background

Affordability and cost considerations are a key barrier to contraceptive access, especially for those with lower incomes. In 2011, 1/3 of adult US women in a nationally representative survey who had ever tried to obtain prescription contraception reported access barriers, with affordability and/or lack of insurance being the most common (Grindlay & Grossman, 2016). Nationally as of 2016, overall contraceptive use among women at risk of pregnancy was about five to seven percentage points lower among women in lower income groups as compared to women with higher incomes (Kavanaugh & Pliskin, 2020), and one in five women would choose a different method of contraception if cost were not a factor in their decision (Burke et al., 2020). In a recent statewide study of reproductive-aged women in Ohio, 25% reported not using their preferred contraceptive method, with affordability being the most common barrier cited (Chakraborty et al., 2021).

Cost-related barriers to contraception can be minimized by either reducing the costs associated with contraceptive care visits or subsidizing contraceptive methods. In the U.S., one key route through which these cost saving techniques have been implemented is through the nationwide network of publicly supported family planning clinics, which is made up of over 10,000 sites across the country. This network provided contraceptive services to 6.2 million women across the US in 2015 (Frost et al., 2017). Many publicly supported providers are recipients of Title X, a federal grant program that provides dedicated funds for sexual and reproductive health care for low-income individuals. In 2019, 64% of patients obtaining care at Title X-funded clinics were living at or below the federal poverty level and 41% were uninsured (Fowler et al., 2020). Thus, the network of publicly supported family planning clinics is crucial to the delivery of sexual and reproductive health (SRH) care for young, low-income, and uninsured people and other marginalized communities.

Health insurance coverage is another key avenue that reduces cost-related barriers for individuals and facilitates access to contraception. Nationally, those with any type of health insurance coverage have higher levels of overall contraceptive use, and those with access to contraceptive care facilitated by insurance coverage had higher odds of using certain provider-controlled methods like IUDs, pills, patches, shots, and rings (Kavanaugh & Pliskin, 2020). State-level evidence mirrors these insurance-driven contraceptive patterns (Kavanaugh et al., 2020). Several studies have demonstrated how the federal contraceptive coverage guarantee in the Affordable Care Act led to increases in both use of prescription contraceptive methods—especially IUDs and implants—as well as more consistent contraceptive use overall (Becker et al., 2021; Lee et al., 2020; Malcolm et al., 2021). Although private insurance is the most common type of coverage among reproductive-aged women receiving contraceptive care in the United States—accounting for 69% of patients in 2015–2019—about 22% of these patients have public health insurance coverage (Frost et al., 2021). Further, Medicaid is the most common type of coverage among those seeking contraceptive care at publicly support family planning sites (Frost et al., 2021).

Given the substantial role of publicly supported family planning health facilities and health insurance coverage in increasing individuals’ access to contraception in the US, Federal and state policies around these funding streams can have an outsized role in facilitating or impeding contraceptive access. A particularly clear example of this occurred in 2012 when Texas legislators cut state funding for family planning services and passed legislation to exclude abortion-affiliated providers from participating in the state Medicaid program (White et al., 2012). As a result, about a quarter of family planning clinics in Texas were forced to close, resulting in significantly fewer patients served (White et al., 2015). In addition, patients were also forced to pay more for contraceptive services that had been reduced in cost or free under the program (Hopkins et al., 2015).

In July 2017, Iowa discontinued participating in the federally run Medicaid family planning program, forfeiting millions of dollars in federal funding. These family planning-specific insurance coverage programs cover a limited set of family planning-related services for individuals who fall within certain income thresholds, and patients often enroll in these programs at the point-of-care or local health departments (Iowa Department of Human Services n.d.). The state instead opted to implement its own state-level family planning program (Gold & Hasstedt, 2017), which excluded any clinics that provided abortion care or referrals from being eligible for funding (Rodriguez & Sanders, 2017). Consequently, patients who had previously had federal family planning Medicaid coverage were automatically transitioned to the newly established state family planning program and, along with all newly enrolled patients on this plan, were not able to access subsidized care at publicly funded clinics affiliated with abortion provision. This resulted in several large health care entities in Iowa losing funding, which led to closure of four specialized SRH care centers (Butz, 2018; Rodriguez & Sanders, 2017). Of these four, three were concentrated in the southeastern part of the state, effectively leaving an entire region of Iowa without this type of specialized SRH care center, driving over 15,000 patients to find a new family planning provider (Levintova, 2017). Since then, there has been a significant drop in the number of Iowans using the family planning program over time, and providers only spent a fraction of the $3 million allotted by the program (Fowler et al., 2019, 2020, 2021; Rodriguez, 2019). One final key shift in funding streams within the publicly funded family planning system occurred in 2019, which has implications for the analysis presented. In August 2019, the “Trump Final Rule,” which prohibited federal Title X funds from being distributed to health care centers that provided abortion-related care, was implemented (Hasstedt & Dawson, 2019). A practical state-level consequence of this federal-level policy was that Planned Parenthood health care centers in Iowa lost Title X federal support beginning in August 2019.

Given the critical role that contraception plays in an individual’s ability to realize reproductive autonomy, it is essential to investigate the impact of changes in Iowa’s policies related to publicly supported family planning services on individual’s ability to access contraception. Thus, we designed the Reproductive Health Impact Study to broadly track and measure the impact of policy-related changes on the publicly supported family planning network and the people who rely on it in Iowa and three other states. This particular analysis aims to fill a gap in the literature by examining the relationship between individuals’ fluctuating access to care in the years after Iowa’s policy shift and changes in their contraceptive use over time. We hypothesized that the 2017 changes to Medicaid coverage in Iowa would lead to disruptions in people’s access to SRH care, especially within healthcare networks and facilities that were most impacted by the policy changes due to the abortion-related care provided at these sites. We further hypothesized that these disruptions in access to SRH care would likely impact individuals’ contraceptive use, both overall and with regard to using preferred contraception. Most existing evidence on the relationship between access to care and contraceptive use is cross-sectional and therefore correlational. In contrast, we exploit a unique panel dataset of individuals who originally sought care at a publicly supported family planning site in Iowa in 2018 and then participated in subsequent follow-up surveys every 6 months for 2 years to examine an effect of access to care on contraceptive use.

## Methods

### Sample and Fieldwork

#### Sampling and Recruitment

Using a database updated to reflect all known publicly funded family planning centers across the US in 2015 (Frost et al., 2017) and then updated to reflect site openings and closures in Iowa through March 2018 based on conversations with Iowa state contacts who work in the family planning field, we identified all sites in Iowa that received public funds to support the delivery of family planning care to 100 or more female patients annually^1^ and were open as of February 1, 2018. Starting in May 2018, study team members reached out to clinic administrators at each of the 42 eligible facilities to seek their site’s participation in recruiting family planning patients into the study. During recruitment, we identified three additional sites across Iowa that reported serving 100 or more patients annually, either because they recently began providing services or had increased their annual caseload since the initial sample identification, resulting in a total of 45 sites eligible for recruitment into the study. Administrators from 23 of the 45 eligible facilities agreed to their site’s participation in the study. One site was unsuccessful in recruiting enough patients to complete the survey, which resulted in a final sample of 22 participating sites.

Study team members worked with clinic administrators to support front-facing health center staff who managed registration or patient intake forms to recruit potentially eligible patients. Front-facing staff were trained to offer a survey to all potentially eligible patients who visited their facility during each sites’ specific fielding period. After describing the study purpose and the voluntary nature of the study, front-end clinic staff distributed one tablet to each willing patient to complete an online eligibility screener and, if eligible, to complete the online survey. Patients were eligible to participate in the study if they were seeking family planning care,^2^ were at least 15 years old, and had not previously taken the survey.

After screening for eligibility, patients initially completed the survey while in the waiting room or in the exam room; we also added an option for patients to provide an email address to complete the survey outside of the clinic setting 6 months after the fielding process began to increase response rates. Respondents who completed the survey were eligible to enter a site-specific raffle to win a $50 gift card. During the final month of data collection to further increase the number of completed surveys, study team members visited select eligible clinics to recruit patients directly and to offer an additional $10 in cash to each respondent after completion. Patients were recruited for an average of 12 weeks during the baseline fielding period of May 2018–February 2019. The average response rate across all sites based on the number of completed surveys and clinic administrators’ counts of eligible patients was 20%, with response rates for individual sites ranging from 4 to 48%. In total, 1448 patients enrolled in the study.

#### Baseline Data Collection and Survey Instrument

The baseline instrument was a self-administered questionnaire formatted for a web-based, handheld tablet. Questionnaire content focused on access and barriers to SRH care, contraceptive use, and pregnancy attitudes and drew items from previous surveys of patients at family planning sites and the statewide Surveys of Women in MD, DE, AL, and SC (Delaware Contraceptive Access Now Evaluation n.d.), among others. Our data management partners at NORC at the University of Chicago programmed the baseline survey instrument, which also included an eligibility screener (see criteria below), on Samsung Galaxy tablets in both English and Spanish in Voxco, a secure state-of the-art Computer Aided Interviewing (CAI) system. After the instrument was programmed, the screener and survey went through rigorous internal testing by NORC and the study team, and it was externally pretested by a total of 19 patients during two in-clinic visits at a publicly supported family planning site in New York City.

Eligible patients who consented to participate following the screener proceeded to fill out the survey, which had a total of 68 questions. We considered a survey to be “complete” if the respondent answered items up to at least 2/3 of the way through the questionnaire where a key question for our analysis was located. Questions were primarily in multiple choice, check-all-that-apply and grid question format, with a few open-ended write-in options included for responses to select items. The survey also included a drop-down list of all known publicly funded family planning sites in Iowa from which respondents could select to indicate where they previously received care. It took on average 25 min for respondents to complete the survey. Once respondents completed the survey, they were able to elect to provide contact information to enter the site-specific gift card raffle or to be contacted regarding follow-up research, or both.

#### Longitudinal Data Collection and Survey Instruments

After completing the baseline survey, respondents were asked to participate in follow-up surveys every 6 months for the 2 years following completion of the baseline survey. Those who opted into participating in these longitudinal surveys were sent a survey link via email and/or the US postal service. Respondents were offered incentives for each follow-up survey, starting at $15 and increasing by $5 with each of the four follow-up surveys. Those who were invited to participate via mail received an initial $5 cash pre-incentive with each initial follow-up survey invitation and the rest of the incentive in the form of an Amazon gift code following completion of the survey. Respondents who were invited to participate over email were provided the entire incentive as an Amazon gift code following completion. Similar to the baseline questionnaire, follow-up survey instruments included questions about contraceptive use and satisfaction, SRH care use and access, and demographic information, among other foci. The follow-up surveys were formatted to be accessible online on the secure Voxco platform on a variety of devices, including mobile phones, tablets, and desktop computers, and they took approximately 10–15 min for respondents to complete.

The research study protocol and addenda for the baseline and longitudinal surveys were approved by our and our data management partner’s respective Institutional Review Boards.

### Analysis

#### Analytic Sample

Of the 22 sites from which we collected 1448 completed baseline surveys, respondents who reported a confirmed pregnancy at baseline (*n* = 2) were assigned male at birth (*n* = 14) or who did not provide meaningful responses to enough questions on the baseline questionnaire (*n* = 2) were deemed ineligible for analysis and removed from the sample. Respondents from an additional five sites were removed from the sample (*n* = 42) because their site of care at baseline served fewer than 100 patients annually based on a 2018 census of publicly funded family planning sites in Iowa conducted by the Guttmacher Institute (*Unpublished tabulations from the Reproductive Health Impact Study 2018 Family Planning Clinic Census* n.d.); this resource became available after baseline recruitment into the study was complete. Of the remaining baseline sample of 1388 respondents from 17 sites, respondents who completed at least one follow-up survey were eligible for inclusion in our analytic sample of 405 respondents from 16 sites (or 29% of the eligible baseline sample).^3^ Notably, this sample represents an unbalanced panel, with 55% participating at all five time points and the rest participating in two-to-four time points. Finally, respondents were removed from the sample if they responded “yes” at any time point to the question “are you currently trying to get pregnant?”; our final analytic sample comprised 368 respondents.

#### Measures

**Fig. 1:**
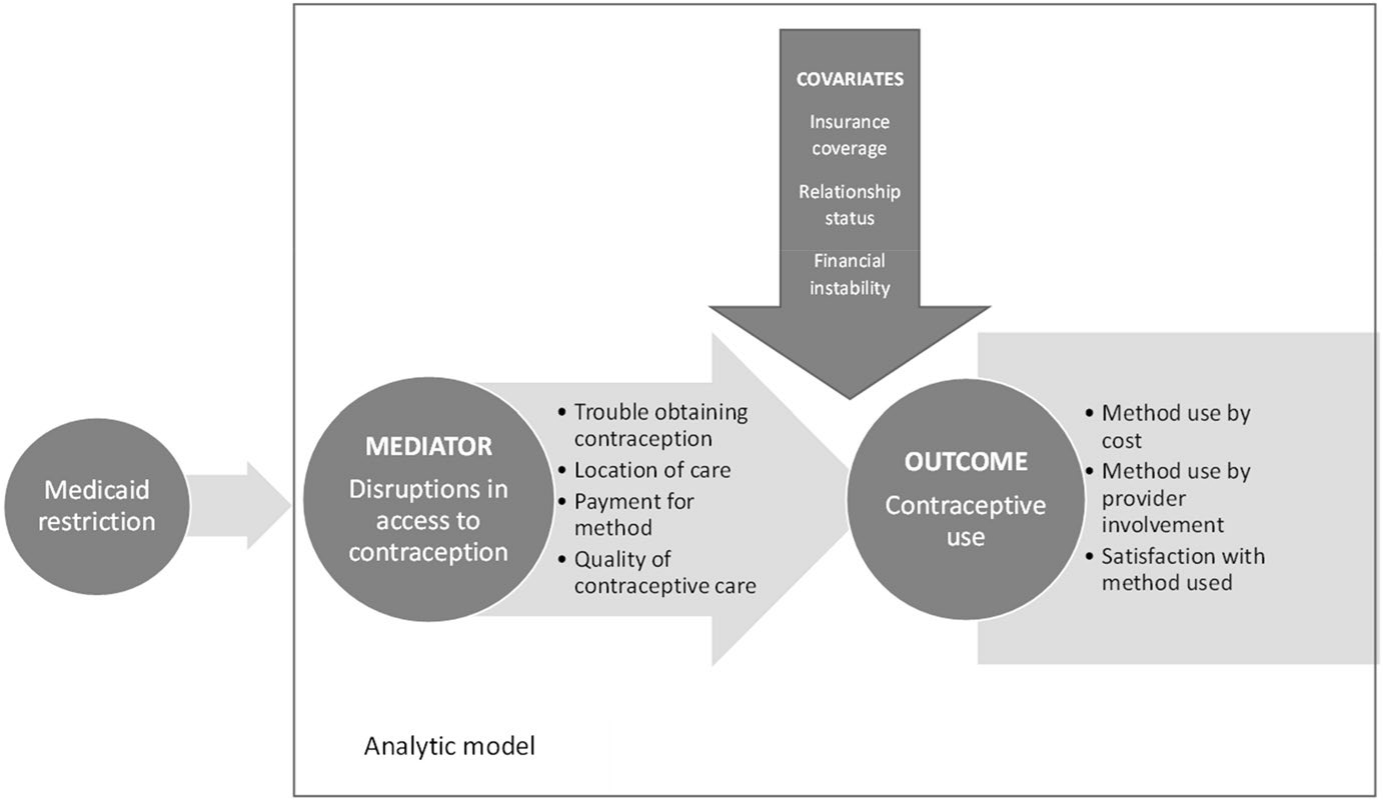
Hypothesized causal pathway from implementation of Medicaid restrictions in Iowa to impact on contraceptive use, with analytic model highlighted within the pathway

We lay out the hypothesized causal pathway through which the implementation of the Iowa Medicaid-related restrictions in 2017 may have led to impacts on contraceptive use in the state (Fig. 1). Our study focuses on the pathway between these two events, specifically disruptions in access to contraception that may have been a result of the changes to the Medicaid program and the subsequent changes in contraceptive use. We operationalize this mediator and outcome in several ways, and we account for time-varying covariates that may have confounded any identified relationships.

#### Mediator: Access to Care

We conceptualized potential disruptions in access to care in four ways: trouble obtaining contraception, location of contraceptive care, payment for contraceptive care, and quality of contraceptive care. Changes in each of these aspects of receiving contraceptive care represent potential disruptions in access for patients. In each survey, respondents were asked about the previous 12 months^4^ (baseline) or 6 months (follow-ups) and whether they had *delayed or had trouble getting their preferred contraceptive method* (yes/no/prefer not to answer) and had received contraceptive care during this timeframe. Those who preferred not to answer were coded as “missing.” Among respondents who reported having received care, we asked them to identify where that care was obtained from a drop-down list of publicly funded family planning sites in Iowa or by writing in the name of their health care site, how they paid for their most recent contraceptive method, and the quality of contraceptive care they had received.

We created an ordinal *location of recent contraceptive care* variable with three categories: no recent care, recent care at a site that may have been impacted by the 2017 Iowa Medicaid restrictions, and recent contraceptive care at a site not likely impacted. These latter two categorizations were determined by whether the site either provided abortion care or referrals or was part of a larger network of care within which abortion-related care was provided (potentially impacted site) or not (non-impacted site) at the time the respondent took the survey. We identified potentially impacted networks of health centers through in-state stakeholder conversations and combined that information with network-level information from our database, which by this time had been updated to reflect all known sites open in 2018 (*Unpublished tabulations from the Reproductive Health Impact Study 2018 Family Planning Clinic Census* n.d.), or the internet (write-in responses only) to categorize respondents’ location of care accordingly. All respondents at baseline were considered to have received care, and clinics where respondents took the baseline survey were similarly categorized according to whether the site was likely impacted by the Medicaid restrictions. Because the baseline timepoint closely aligned with the timeframe asked about in the survey item at the first 6-month follow-up, these baseline categorizations were also used to categorize care for respondents at the first 6-month follow-up survey who reported not receiving care at a clinic in the last 6 months. With the exception of this first, 6-month follow-up time point, respondents who did not report receiving recent contraceptive care at a clinic at baseline or all other follow-up surveys were coded as “missing” for all further time points.

We created a dichotomous variable to represent *payment for contraceptive care* drawing on two separate items that asked respondents how they had paid for their most recent contraceptive method^5^ and provider visit related to contraception, with respondents who indicated that they had paid some or all of either of these contraceptive care costs themselves (including any insurance co-pays) as “yes” and all other payment categories coded as “no.” Anyone who did not report having received either a contraceptive method or care in the last 6 months (for each follow-up survey) or 12 months (for the baseline survey) or who said “prefer not to answer” to both of these payment questions is coded as “missing.”

Our measure of *quality of contraceptive care* was based on the person-centered contraceptive counseling (PCCC) metric. The PCCC includes four items asking respondents to rate their most recent contraceptive provider on a Likert scale: respecting the respondent as a person, letting the respondent say what mattered to them about birth control, taking the respondent’s preferences about their birth control seriously, and giving the respondent enough information to make the best decision about their birth control (UCSF Person-Centered Reproductive Health Program, 2021). Following best practices laid out by the team who developed the PCCC (Dehlendorf et al., 2021; “Using the measure” n.d.), we combined these variables by coding those who rated their provider as “excellent” on all four characteristics as “yes,” meaning they did receive person-centered contraceptive care, and by coding those who rated their provider as anything less than excellent on at least one characteristic as “no.” Those who said “prefer not to answer” to all four PCCC questions were coded as “missing.” Additionally, anyone who did not receive any form of care in the past 6 months (for each follow-up survey) or 12 months (for the baseline survey) were coded as “missing.”

#### Outcome: Contraceptive Use

Contraceptive use was conceptualized in three ways that may be sensitive to disruptions in access: contraceptive use according to cost, contraceptive use according to provider involvement, and satisfaction with contraception. In each survey, respondents were asked whether they used any method or methods of contraception, even for reasons other pregnancy prevention, in the past 3 months. Those who indicated “yes” were presented with a list of contraceptive methods to indicate which method or methods they had used. Those who indicated “no” made up our “no use” category for all outcome variables. Finally, those who reported not using a method in the last three months were asked on a separate item to report their reasons for not using any method of birth control; those who reported “we just use the pull out method” or who wrote-in reasons for not using that implied permanent method use were recoded to be method users.

We created an ordinal *contraceptive use according to cost* variable, with three categories: no use, use of contraceptive method that does not carry cost to the individual (including withdrawal, natural family planning methods, abstinence during sexually active periods, and partner vasectomy), and use of a contraceptive method that may carry cost to the individual (including pills, the patch, the ring, Depo-Provera®, condoms, other barrier methods, and emergency contraception). Our ordinal *contraceptive use according to provider involvement* variable was similarly created, with condoms, other barrier methods, and emergency contraception moving from the top-coded category to the middle category when considering a provider’s role in an individual’s access to these methods. For respondents who indicated use of IUDs or implants, we categorized use of these methods at baseline as not carrying cost or requiring provider involvement and all subsequent initiation of use of these methods as carrying cost and potentially requiring provider involvement. For respondents who indicated use of one’s own permanent contraceptive method (e.g., tubal ligation and hysterectomy) at baseline, we categorized this method use as carrying no cost and requiring no provider involvement. For all first-time reports of IUDs, implants or respondents’ own permanent contraceptive method use in follow-up surveys, we assumed they recently initiated the method and categorized use at this time point as carrying cost and potentially requiring provider involvement. Respondents who reported “yes” to all methods, “no” to all methods, or “prefer not to answer” to all methods were coded as “missing” for both items.

Finally, we created an ordinal *satisfaction with contraception* variable, with three categories: no use, not satisfied with contraceptive method used, and satisfied with method used. We combined an item measuring method satisfaction on a Likert scale at baseline with an item asking whether respondents were using their preferred method in the follow-up surveys. We categorized respondents as being not satisfied with their contraceptive method if they said they were neutral, dissatisfied, or very dissatisfied at baseline or if they indicated that they would prefer to be using a different method in any follow-up survey. We categorized respondents as being satisfied with the method being used if they indicated that they were satisfied or very satisfied at baseline or if they indicated that they were using their preferred method in any follow-up survey. Respondents who were recoded as a method user after reporting no method use but relying on withdrawal were skipped out of the satisfaction measure at baseline so are “missing” on this item.

#### Covariates, Demographic, Socioeconomic, and Baseline Site Characteristics

To describe our analytic sample, we document the site where respondents received SRH care at the baseline time point in terms of whether the site received Title X grant funding to support provision of family planning care and in terms of whether the site may have been impacted by the 2017 Iowa Medicaid restriction or not (described above). The Title X status of the site was determined by triangulating the date of survey completion with our own internal data source that identifies both whether and when every publicly supported family planning service delivery site in the state that serves more than ten patients annually as of 2018 was a recipient of the Title X federal family planning program (*Unpublished tabulations from the Reproductive Health Impact Study 2018 Family Planning Clinic Census* n.d.).

We also present several respondent demographic characteristics, including mean age, income, race and ethnicity, and educational attainment, which were asked only at baseline, and sexual identity, relationship status, insurance coverage and a measure of financial instability, each of which were asked at every survey timepoint.

Respondents were asked to identify all race options that applied to them among options of Black or African American, White, Asian or Asian American, Native American, Alaska Native, or American Indian, Native Hawaiian or Pacific Islander, and were offered an “other” write-in option, as well as an option to indicate whether they identified as Hispanic or non-Hispanic. Because most of the sample (74%) indicated a non-Hispanic white identity, we collapsed responses, including write-in responses, to create a dichotomous non-Hispanic white (yes/no) variable. To assign respondents’ educational attainment, we created a three-category variable that collapsed respondents’ reported highest level of education attained into less than or equal to a high school diploma or equivalent, some college or completion of an Associate’s degree, and college degree or higher. To generate an income as a percentage of federal poverty level variable, we identified household income categories by combining write-in responses to an item asking total household income for 2017 (the year prior to the survey) with responses to a categorical probe from those who did not respond to the write-in. Using the US Department of Health and Human Services federal poverty guidelines for 2017 (US. Department of Health and Human Services under the authority of 42 U.S.C. 9902(2) 2017), we combined this information with respondents’ income category and reported household size to assign income as a percentage of poverty. We then imputed missing values to align the sample with the population universe by patient characteristics (see details above).

Respondents were asked to select the sexual identity that best aligned with their experience from a list including lesbian or gay, straight, bisexual, pansexual, or “something else,” which allowed respondents to write-in a description of their identity. Almost 80% of the sample identified as straight, so we combined all responses other than “straight,” into one category to create a binary sexual identity variable, with all write-ins recoded to the most relevant category. We created a three-category relationship status variable combining respondents’ answers to two variables: one asking whether they live with a romantic partner and a second asking for respondents’ formal marital status including now married, separated, widowed, divorced, and never married. To create the relationship status recode, we prioritized those who were currently married, followed by those who were not married and living with a romantic partner, and finally those not married and not living with a romantic partner.

We also asked respondents at each time point to identify all types of insurance coverage they currently had from a list. We created a three-category variable to condense these responses into no insurance, private health insurance coverage (employer-based coverage and coverage purchased on the marketplace), and public health insurance coverage (including Medicaid, Medicare, Indian Health Services coverage, TriCare, and the State Family Planning Program formerly the Iowa Family Planning Network). Respondents who selected “no” to all insurance coverage options were coded as not having insurance. Respondents who selected “yes” to all options, “don’t know” to all options, “prefer not to answer” to all options or reported another type of insurance not listed were coded as “missing.”

Finally, we include a measure representing respondents’ economic situation that may relate to both their access to care and contraceptive use over time: experience of recent financial instability at each survey time point. This dichotomous item asked respondents whether they had fallen behind on their rent or mortgage in the past 12 months (baseline) or 6 months (follow-ups). Those who indicated “yes” were considered to have experienced financial instability at that survey timepoint.

#### Analytic Strategy

We first conducted descriptive analyses of our sample. We conducted supplementary analyses to compare tabulations of key demographic characteristics of the eligible analytic sample (405) to those for three subpopulations of the overall study sample: the full study population who completed the baseline survey (1388), the population who opted-in to participating in the follow-up surveys but who did not complete any (983), and the population who completed the baseline survey but who did not opt-in to participating in the follow-up surveys (767).

We then present tabulations of our key mediators, outcomes, and covariates at each timepoint over the study period for the analytic sample, including chi-square tests to demonstrate whether each of these variables changed significantly over the full study period. To further illustrate changes in key variables over the study period, we plotted overall shifts in respondents’ location of recent contraceptive care according to whether the site may have been potentially impacted by state Medicaid restrictions, within-respondent shifts in location of care over study period, and shifts in contraceptive method use according to cost over the study timeframe. When examining overall shifts in respondents’ location of care and contraceptive method use, we present findings among all respondents and among respondents reporting public health insurance coverage at any time point during the study. We focus on this latter population group due to the potential that this group may have been most sensitive to impacts of Medicaid-related coverage restrictions linked to site of care.

Finally, we estimated ordinal conditional logistic regression models that controlled for observed (measured within datasets) and unobserved (not measured) non-changing characteristics within individuals to examine respondent-level fixed effects of our key independent variables related to contraceptive access—trouble obtaining contraception, location of recent contraceptive care, payment for contraceptive method, and quality of contraceptive care—and our three outcomes of interest (contraceptive use according to cost, contraceptive use according to provider involvement, and satisfaction with contraception) (Fig. 5). In each model, we controlled for key time-varying sociodemographic variables known to have an impact on contraceptive use: relationship status, recent financial hardship, and health insurance coverage. All analyses were performed within Stata version 17.0. The regression models account for the clustered nature of our sample design. Adjusted odds ratios and 95% uncertainty intervals are presented for each of the models, and we highlight findings significant at *p* < 0.1 below. Tables presenting more detail from the models are included in the Appendix.

## Results

### Sample Characteristics

Most of the 368 respondents in the analytic sample were recruited into the study when they sought family planning care at a site that received public support for these services through Title X (76%), and over four-fifths (83%) were recruited at a site potentially impacted by the Medicaid coverage changes in 2017 (Table 1). Respondents’ average age at the baseline survey was 26 years old, and about one-third of the sample fell into each of the three income categories. About ¾ of the sample identified as non-Hispanic white, 12% identified as Hispanic, and 7% each identified as non-Hispanic Black or another non-Hispanic racial identity. The majority of the sample identified as straight (79%). Half had received some college education or an Associate’s degree, and the remaining half was split about equally between having a high school degree or less (24%) or a college degree or higher (26%).^6^

**Table 1:** Demographic characteristics of Iowa family planning patients ages 15 + at baseline in analytic sample, (2018–2019)

	Baseline sample
	%
Total (*N*)	368
Received care at a Title X site at baseline	
No	24%
Yes	76%
Received care at a facility potentially impacted by Medicaid restrictions at baseline	
No	17%
Yes	83%
Age, mean years (standard deviation)	25.8 (7.7)
Income as a % of the federal poverty level	
< 100%	35%
100–199%	32%
200% +	33%
Race and ethnicity	
White non-Hispanic	74%
Black non-Hispanic	7%
Other non-Hispanic	7%
Hispanic	12%
Sexual identity	
Not straight	21%
Straight	79%
Educational attainment	
< HS degree/HS grad or GED	24%
Some College/Associates	50%
College grad or more	26%

Respondents were included in the analysis if they received family planning care at baseline from a publicly supported health care center that served 100 or more family planning patients in Iowa in 2018, if their sex assigned at birth was female, if they did not have a confirmed pregnancy at baseline, if they completed the baseline survey and at least one follow-up survey, and if they did not report trying to become pregnant at every survey time point. Some characteristics do not sum to 100% due to nonresponse

### Changes in Key Variables Over Time

**Fig. 2:**
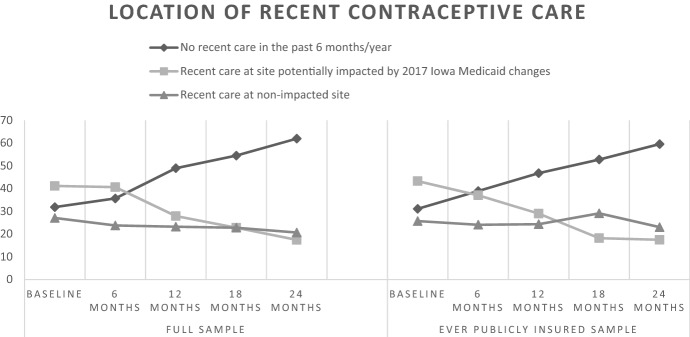
Location of recent contraceptive care over study period according to whether the site may have been potentially impacted by Iowa Medicaid restrictions among all respondents and among respondents reporting public health insurance coverage at any time point during the study, omitting baseline care site

Time-varying responses that are the focus of our analyses are shown in Table 2. Of the 368 baseline respondents, between 272 and 317 respondents completed the follow-up surveys (biannuals 1, 2, 3, or 4). Over the time period, 80% of respondents reported shifting their receipt of recent contraceptive care between three possible care options, with respondents increasingly reporting having received no recent contraceptive care, from 32% at baseline up to 62% at the end of the study (*p* < 0.001).^7^ Simultaneously, respondents less frequently reported having received recent care at a site potentially impacted by the Medicaid restrictions, with 42% reporting this outcome at baseline and only 17% reporting this location of care at the final wave. Similar patterns in location of contraceptive care were observed among those respondents who reported being covered by public insurance at any time during the study period, with those getting care at a non-impacted site staying somewhat steadier over the time period (Fig. 2).

**Table 2:** Distribution of mediator, outcome, and covariate variables from survey responses at each timepoint over 2-year study period, 2018–2021

	Baseline (4/18–1/19)		6 months (10/18–7/19)		12 months (4/19–2/20)		18 months (10/19–8/20)		24 months (4/20–2/21)		Any change in metric over study period (4/18–2/21)		
	*N* = 368		*N* = 272		*N* = 292		*N* = 284		*N* = 317		*N* = 368		
	*N*	%	*N*	%	*N*	%	*N*	%	*N*	%	*N*	%	*p* value^e^
Mediators													
Location of recent contraceptive care^a^											295	80.2	< 0.001
No recent care in the past 6 months/year	113	31.8	0	0.0	135	48.9	153	54.4	192	61.9			
Recent care at site potentially impacted by 2017 Iowa Medicaid changes	146	41.1	192	70.6	77	27.9	64	22.8	54	17.4			
Recent care at non-impacted site	96	27	80	29.4	64	23.2	64	22.8	64	20.6			
Paid out-of-pocket for a contraceptive method or related care^b^											159	43.2	0.10
No	214	72.5	162	73.3	153	78.1	144	80.0	120	69.0			
Yes	81	27.5	59	26.7	43	21.9	36	20.0	54	31.0			
Trouble getting contraception in past (6 months/year)											137	37.2	0.01
No	286	78.1	234	86.3	200	84.0	198	84.6	223	88.1			
Yes	80	21.9	37	13.7	38	16.0	36	15.4	30	11.9			
Quality of contraceptive care experienced^b^											150	40.8	0.09
Less than excellent	61	21.0	64	29.2	57	29.4	49	27.4	37	21.4			
Excellent	230	79.0	155	70.8	137	70.6	130	72.6	136	78.6			
Outcomes													
Use of contraceptive method according to cost^c^											177	48.1	< 0.001
No use	32	8.9	24	9.1	27	9.8	38	13.7	47	15.2			
Use of no cost method	55	15.3	37	14.0	53	19.2	69	24.9	85	27.5			
Use of method that carries cost	273	75.8	204	77.0	196	71.0	170	61.4	177	57.3			
Use of provider-involved contraceptive method^c^											172	46.7	< 0.001
No use	32	8.9	24	9.1	27	9.8	38	13.7	47	15.2			
Use of non-provider-involved method	136	37.8	71	26.8	92	33.3	103	37.2	128	41.4			
Use of provider-involved method	192	53.3	170	64.2	157	56.9	136	49.1	134	43.4			
Satisfaction with contraceptive method used^c^											223	60.6	0.02
No use	32	9.2	24	8.8	27	9.2	38	13.4	47	14.8			
Not satisfied with method being used	63	18.1	63	23.2	79	27.1	70	24.6	69	21.8			
Satisfied with method used	254	72.8	185	68.0	186	63.7	176	62.0	201	63.4			
Time-varying covariates													
Relationship status											140	38.0	0.04
Married	36	9.9	32	11.8	31	10.7	40	14.2	42	13.3			
Neither married nor cohabitating	236	65.2	150	55.4	168	58.1	149	52.8	164	51.9			
Cohabitating	90	24.9	89	32.8	90	31.1	93	33.0	110	34.8			
Insurance status^d^											129	35.1	0.30
No insurance	38	10.8	27	10.1	25	8.8	21	7.6	23	7.3			
Private insurance	203	57.8	167	62.3	193	67.7	180	65.0	199	63.6			
Public insurance	110	31.3	74	27.6	67	23.5	76	27.4	91	29.1			
Experience of recent financial instability											129	35.1	0.03
No	282	77.0	219	80.5	244	83.8	244	86.2	262	82.9			
Yes	84	23.0	53	19.5	47	16.2	39	13.8	54	17.1			

^a^Excludes those who got recent care but not at a clinic. The full sample received recent care at the first 6-month follow-up survey time point. We considered any respondent who did not directly report having received recent care at that 6-month follow-up to have received recent care because they had received care at the baseline facility where they enrolled in the study, which occurred approximately 6 months prior to completing the first 6-month follow-up survey

^b^Among respondents who received a contraceptive method or related service in the past 6 months/year

^c^Among respondents who had sex in the last 3 months

^d^Private insurance includes employer‐based plans and plans purchased on the marketplace or exchange. Public insurance options include Medicaid, Medicare, Tricare, Indian Health Service and Iowa's state funded family planning program.

^e^*p* values from chi-square tests determining level of significant change in proportions across any of the survey time points

**Fig. 3:**
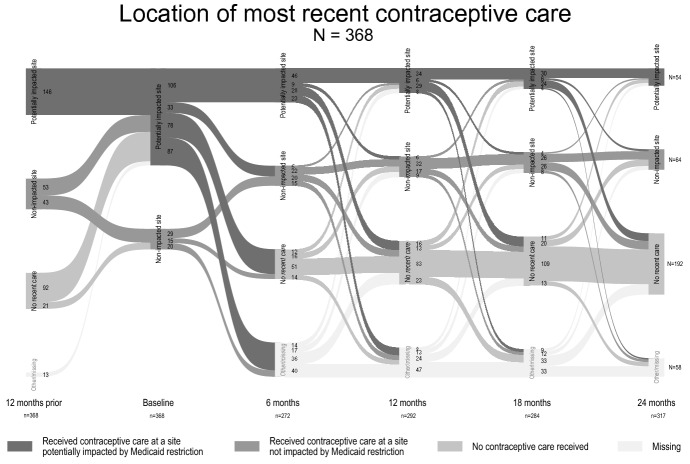
Sankey diagram of within-respondent change in location of recent contraceptive care over study period according to whether the site may have been potentially impacted by Iowa Medicaid restrictions

Respondents’ movement over the study period between different locations for recent contraceptive care, including the baseline survey site as a distinct time point for care, is presented in Fig. 3. Most respondents (83%) took the baseline survey at a potentially impacted health care site, and about half of these (48%) reported receiving SRH care at this same type of site in the previous year. The remaining respondents who took the baseline survey at a potentially impacted site had either received SRH care at a non-impacted health care site (17%), had not received any SRH care (30%), or did not respond to the survey item about SRH care in the past year (4%). Over the subsequent four timepoints during the study period, the percentage of respondents indicating that they had received SRH care in the previous 6 months at a potentially impacted site steadily decreased from 29% at the six-month follow-up to 21% at 1 year to 17% at 18 months to 15% at 24 months. At the same time, the percentage of respondents indicating having received recent SRH at a non-impacted site remained relatively steady (17–18% across waves) and those indicating having received no recent SRH care increased steadily from 25% at the 6-month follow-up to 37% at 1 year to 42% at 18 months to 52% at 24 months.

Respondents’ reports of recent payment for contraceptive care fluctuated over the study period, starting at 28% at baseline, decreasing to about 20% over the subsequent 18 months, and then increasing to 31% by the end of the study (Table 2). Overall, 43% of the sample reported shifting within these payment categories over the study period, but these changes were only marginally significant (*p* = 0.1). Reports of having any recent trouble obtaining preferred contraception were relatively low over the entire study period and decreased from 22% at baseline to 12% at the final wave, with about 37% of respondents indicating any change in this experience (*p* = 0.01). Reports of experiencing person-centered contraceptive care were common among the sample over the full study time period, with between 71 and 79% reporting this experience across the waves. Just over 2/5 of the sample shifted marginally between experiencing person-centered to non-person-centered care during the study period (*p* = 0.09).

**Fig. 4:**
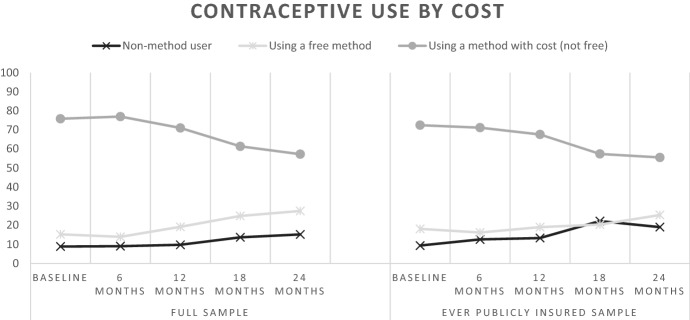
Use of contraceptive method according to cost over study time frame among all respondents and among respondents reporting public health insurance coverage at any time point during the study

Over the study period, nonuse of contraception increased from 9% at baseline to 15% at the final wave. When considering contraceptive method according to cost of the method, use of a method that carries a cost decreased from 76% at baseline to 57% at the final wave, while use of a no cost method increased during this same time frame from 15 to 28% (Fig. 4). About half of the sample shifted within these contraceptive cost categories over the study period (*p* < 0.001). Among respondents who reported ever having public health insurance coverage, nonuse increased from 9% at baseline to 19% by the end of the study. Over the same time period, use of a method that carries cost decreased from 72 to 56% and use of a free method increased slightly from 18 to 25%.

Similarly, when considering contraceptive use according to provider involvement, provider-involved contraceptive use decreased overall from 53% at baseline to 43% at the final wave (Table 2). Use of non-provider-involved methods fluctuated somewhat, remaining around 2/5 of respondents between baseline and the final wave. Just under 1/2 of the sample shifted within these provider-involved contraceptive categories over the study period (*p* < 0.001). Satisfaction with one’s method decreased overall during the study time frame, from 73% at baseline to 63% by the end, while dissatisfaction overall remained relatively steady at around 1/5 of the sample from beginning to end. Almost 3/5 of the sample changed their satisfaction level with their current contraceptive method over the study period (*p* = 0.02).

In terms of time-varying respondent characteristics examined in association with our outcomes, two of the three covariates demonstrated significant change over the study period. Most commonly, respondents were neither married nor cohabiting and this decreased somewhat over the study period (65% at baseline and 52% at the final wave), while the proportions who were cohabiting and married increased somewhat (from 25 to 35% and 10–13%, respectively, *p* = 0.04). Respondents reported high levels of overall health insurance coverage at baseline (only 11% were uninsured), and private insurance was more common than public insurance (58% vs. 31%). There was no significant change in health insurance coverage over the full study timeline. Finally, the majority (77–83%) reported no financial instability at baseline and the final time point; between 14 and 23% did report financial instability at some point during the study period (*p* = 0.03).

### Respondent-Level Associations Between Access to Care and Contraceptive Use Over Time

**Fig. 5:**
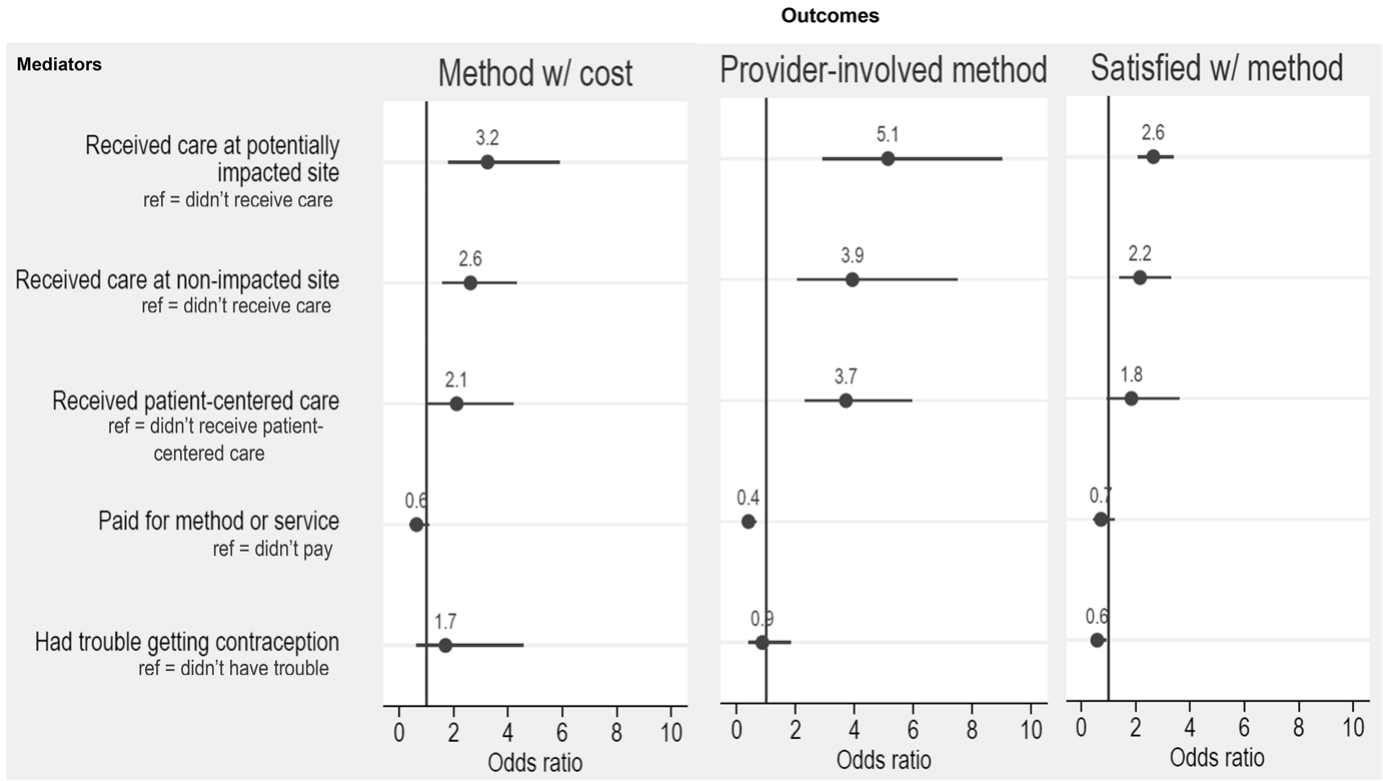
Adjusted odds ratios and uncertainty intervals of the relationship between changes in access to care (mediator variables) and contraceptive use (outcome variables) over the study period. All models control for union type, financial instability, and insurance coverage status

After controlling for all time-invariant sociodemographic characteristics as well as the time-varying variables of relationship status, insurance coverage status, and financial hardship, those who shifted from receiving no recent contraceptive care to having received recent contraceptive care at a potentially impacted site had a 3.3 increased odds, and those who shifted to receiving care at a non-impacted site had a 2.6 increased odds, of shifting to using a contraceptive method that carries cost (*p* < 0.001) (Fig. 5).

A similar association was found between location of recent contraceptive care and use of a provider-involved contraceptive method and satisfaction with contraceptive method used. Those who shifted from receiving no recent contraceptive care to having received recent contraceptive care at a potentially impacted site had 5.1 increased odds, and those who shifted to receiving care at a non-impacted site had a 3.9 increased odds, of using a provider-involved contraceptive method (*p* < 0.001). Those who shifted from receiving no recent contraceptive care to having received recent contraceptive care at a potentially impacted site had a 2.7 increased odds, and those who shifted to receiving care at a non-impacted site had a 2.2 increased odds, of being satisfied with the contraceptive method used (*p* < 0.001). In other words, within this sample, moving from getting no care to getting any care (regardless of the site’s potentially impacted status) increased the likelihood of moving toward using a provider-involved method, a method that carries cost, and being more satisfied with one’s method.

Among those who had received recent SRH care over the study period, respondents who shifted from not experiencing person-centered care to having that care had increased odds of shifting to using a method that carries cost (aOR = 2.1, *p* = 0.03) and a provider-involved method (aOR = 3.7, *p* < 0.001). There was a marginally significant association between changes in respondents’ experience of person-centered care and changes in their satisfaction with their method (aOR = 1.8, *p* = 0.07).

Respondents who shifted from not paying any out-of-pocket costs for contraceptive care (visit or methods) to paying something had 0.4 decreased odds of shifting to using a provider-involved contraceptive method (*p* < 0.001). There was no significant association between changes in respondents’ out-of-pocket payments for contraceptive care and methods and changes in their contraceptive use according to cost or satisfaction with their method.

Finally, those who moved from reporting no recent trouble accessing preferred contraception to experiencing recent trouble doing so over the study period had a 0.6 decreased odds of being satisfied with the contraceptive method used (*p* = 0.02). There was no significant association between changes in respondents’ trouble accessing preferred contraception and changes in their contraceptive use according to method cost or provider involvement.

## Discussion

We sought to understand how the 2017 Iowa policy that prohibited the use of Medicaid to cover care at health care centers affiliated with abortion provision impacted people’s access to SRH care and their subsequent contraceptive use. During the study period of 2018–2020, Iowa family planning patients in our sample shifted to lower levels of receiving SRH care. These shifts occurred to a greater extent among patients who had initially received care at sites potentially impacted by the changes to Medicaid policy in Iowa than among those who had received care at sites not impacted by the policy changes. At the same time, these patients also shifted toward higher levels of contraceptive nonuse; among the remaining users, use of a contraceptive method that carries cost, use of a provider-involved method, and satisfaction with one’s method decreased. Secondarily, those who shifted toward receiving contraceptive care—either at a site potentially impacted by the 2017 Iowa Medicaid policy restriction or at a non-impacted site—experienced shifts toward using contraceptive methods that carry cost, provider-involved methods and being more satisfied with the method used. Those who shifted toward paying out-of-pocket for their contraceptive care shifted away from using contraceptive methods that carry cost and provider-involved methods. We interpret these findings as preliminary descriptive evidence demonstrating that disruptions in access to contraceptive care are associated with contraceptive outcomes. These relationships are especially notable given the sample’s high levels of access to subsidized care via health insurance and baseline receipt of publicly supported family planning care.

We considered disruptions in access to SRH care in several ways: location of care, payment for care, direct report of experiencing trouble accessing care, and quality of care. Each of these represent important considerations for ensuring person-centered and equitable contraceptive care (Holt et al., 2020). In our study, disruptions in access to SRH care based on changing the location of care were most salient in terms of experiencing the greatest amount of flux over the study period and in being associated with all three contraceptive outcomes examined. Importantly, while most individuals in our study shifted between different sources of SRH care over time, those who had sought care in the beginning of the study at a site where the 2017 Medicaid policy would have restricted its use for SRH care had largely moved to either seeking care at sites where this policy would not have played a role or had not sought SRH care at all by the end of the study. As the largest financier of publicly funded family planning care nationally, and with 16–19% of Iowan women ages 19–64 covered through the program in 2017 when the restrictive policy examined in this study was enacted (The Henry J. Kaiser Family Foundation 2019), coverage through Medicaid stands as a key pathway through which barriers to SRH care, especially for low-income individuals who rely on publicly supported family planning care, can be decreased. Our study provides evidence that this important objective is not being met in the wake of the 2017 Medicaid restrictions.

We examined how these disruptions in access to SRH care led to changes to contraceptive use, conceptualized in three ways to represent important considerations that individuals weigh when selecting a contraceptive method: method costs, provider involvement, and satisfaction with a method (Gomez & Clark, 2013; Lessard et al., 2012; Samari et al., 2020). In our study, impacts on contraceptive use according to method costs and provider involvement due to disruptions in access were more prominent than on method satisfaction. This third operationalization of contraceptive use is arguably the most person-centered outcome of the three examined, and more research is needed to understand how disruptions in access to SRH care beyond changing location of care inhibit individuals’ ability to enact their contraceptive preferences. However, preliminary evidence at the national level indicates that receiving person-centered care is one pathway through which individuals can realize their contraceptive preferences (Kavanaugh et al. n.d.), and state-level initiatives to increase contraceptive access should prioritize these patients’ experiences.

Our study findings contribute to growing evidence that barriers to SRH care, either broadly or with regards to a specific type of SRH care, threaten people’s ability to realize reproductive autonomy (American Public Health Association, 2015). The 2017 Iowa policy likely increased the cost burden of SRH care for patients who had previously sought care at sites potentially impacted by the policy change prior to its implementation, including many specialized reproductive health care sites, which many women rely on and prefer for their primary source of SRH care (Frost et al., 2012). Over time, those who incurred costs for SRH care or contraceptive methods that had previously been free or low cost shifted away from using more expensive methods that require contact with the health care system, often methods that users indicate preferring when cost is not a consideration in contraceptive choice (Chakraborty et al., 2021). Taken in tandem with early evidence from Iowa that patient caseloads at publicly funded family planning healthcare sites fell (Rodriguez, 2019) while abortion (Leys, 2021) and STI rates (Levintova, 2017) increased all following the Medicaid changes, these findings highlight the importance of shoring up financial support systems to reduce patients’ cost burden in order to sustain sexual and reproductive health and autonomy.

Our findings focused on Iowa SRH patients support the broader evidence base around cost being a key barrier to contraceptive access. Cross-sectional studies have highlighted the link between insurance coverage and most/moderately effective methods, most of which are provider-controlled (Culwell & Feinglass, 2007; Frost & Darroch, 2008; Kavanaugh & Pliskin, 2020; Kavanaugh et al., 2020). Several studies have described the impact of the ACA contraceptive coverage guarantee on both reduced out-of-pocket costs for contraception (Frederiksen et al., 2020; Sonfield et al., 2015) and increases in contraceptive use, especially in use of more expensive and effective methods like the IUD. (Becker et al., 2021; Malcolm et al., 2021) All of this evidence highlights the critical role that supportive funding and payment strategies through subsidized SRH care and comprehensive health insurance coverage for this care play in individuals’ ability to realize reproductive autonomy (Coalition to Expand Contraceptive Access, 2021; Guttmacher Institute, 2021).

Our study has limitations that are important to note. Given some key differences between the universe and sample of publicly funded family planning sites in Iowa, generalizability of these findings to the full universe in Iowa, especially as they relate to sites potentially impacted by the state Medicaid coverage changes, should be done with caution. Although we designed the study to achieve a sample size that would be powered to detect differences in our key outcome of contraceptive use, we did not achieve our target sample size due to a combination of recruitment challenges, the number of eligible sites in IA, the caseload of patients seen at eligible sites, and relatively low response rates at several sites within our sample. This limits the extent to which we were able to detect additional differences in our contraceptive use outcomes—especially with regards to method satisfaction—but it does not take away from the key differences that we did detect. In addition, lower levels of change in our mediator variable of experiencing recent trouble getting contraception as compared to change in our other mediators may have impacted the extent to which our models were able to detect key associations between this variable and our outcomes. Respondents categorized as not having received recent contraceptive care at any survey timepoint may have not necessarily had a need for this care during the timeframe; interpreting changes in this outcome as representing changing levels of barriers should be done with caution.

Our study was originally designed to capture impacts on patients related to a 2017 Iowa Medicaid policy. Due to the delayed fielding period relative to the policy implementation, some health centers delivering publicly funded family planning care had already closed prior to the start of our study (Butz, 2018; Rodriguez & Sanders, 2017), indicating that impacts on access had already started prior to our data collection. This may have dampened the impact of the policy observed in our study. In addition, during the study period, two notable events occurred, which have implications for our study findings. The August 2019 implementation of the “Trump Final Rule,” caused the funding status of the remaining Planned Parenthood health care centers in Iowa, including several where patients in our sample had received health care, to shift in the middle of the study period. The second event was the COVID-19 pandemic, which began impacting patients’ access to SRH care toward the end of the study period. Both of these events disrupted access to SRH care during our study period, although we are unable to disentangle the absolute contribution that each of them made to patients’ overall disruption in access to SRH care. However, our data point to shifts in access and contraceptive use in Iowa beginning prior to the 2019 Final Rule going into effect, indicating that the additional limitations placed on family planning providers in 2019 and 2020 may have only exacerbated some of the earlier challenges identified with these findings. Finally, our research question focused on understanding how patients’ changing access to sexual and reproductive health care was associated with changes in their contraceptive use; given our site of recruitment at a healthcare site that receives public support for delivering family planning care, our sample does not include those individuals who were not able to access this (or any) care during the study recruitment period, and who may be the ones who would have experienced the greatest negative impact in terms of reduced access to SRH care and subsequent shifts in their contraceptive use.

## Conclusion

Evidence demonstrating impacts of disruptions in access to SRH care on contraceptive use highlights threats to individuals’ reproductive autonomy, especially for those with low incomes who are the majority of patients seeking care at publicly supported family planning sites. State and federal policy initiatives that impede or restrict broad access to contraceptive services, such as the Iowa Medicaid shifts in 2017 and the changing Title X regulations in 2019, can have real and direct implications for the sexual and reproductive wellbeing of individuals who rely on publicly supported contraceptive care. Our study supports the growing body of literature that supportive payment and funding strategies for contraception enable people to access the contraception they need, thus contributing to their ability to realize reproductive autonomy.

## Data Availability

The data that support the findings of this study are available on request from the corresponding author [MK]. The data are not publicly available due to their sensitive nature.

## Appendix

(See Table 3).

**Table 3:** Adjusted odds ratios and uncertainty intervals from fixed-effects ordinal logistic models assessing the relationship of changes in mediators with changes in outcomes over study period, 2018–2021

	Outcomes								
	Use of a method that is not free			Use of a provider-involved method			Satisfaction with method used		
	aOR	*p* value	Uncertainty Intervals	aOR	*p* value	Uncertainty Intervals	aOR	*p* value	Uncertainty Intervals
Mediators									
Location of recent contraceptive care^a^									
Observations	690			664			885		
No recent care in the past 6 months/year	1			1			1		
Recent care at site potentially impacted by 2017 Iowa Medicaid change	3.247	< 0.001	(1.791, 5.887)	5.14	< 0.001	(2.930, 9.019)	2.648	< 0.001	(2.074, 3.381)
Recent care at non-impacted site	2.618	< 0.001	(1.590, 4.309)	3.93	< 0.001	(2.065, 7.479)	2.16	< 0.001	(1.414, 3.301)
Paid out-of-pocket for contraceptive method or related care^b^									
Observations	331			325			520		
Yes v No	0.634	0.092	(0.373, 1.078)	0.396	0.001	(0.235, 0.670)	0.722	0.222	(0.428, 1.217)
Trouble getting contraception in past (6 months/year)									
Observations	495			498			715		
Yes v No	1.699	0.293	(0.632, 4.570)	0.868	0.712	(0.411, 1.836)	0.582	0.015	(0.377, 0.899)
Quality of contraceptive care experienced^b^									
Observations	329			324			512		
Excellent vs Less than excellent	2.11	0.034	(1.060, 4.203)	3.711	< 0.001	(2.312, 5.956)	1.841	0.073	(0.945, 3.587)

All models control for union type, financial instability, and insurance coverage status

^a^Excludes those who got recent care but not at a clinic. Includes baseline care site as recent care at the 6-month follow-up survey if respondents indicated having received no recent care

^b^Among respondents who received a contraceptive method or related service in the past 6 months/year

## Footnotes

1 Based on 2015 data, which was the most recent year for which these data were available.

2 Family planning care includes receiving a method of birth control, birth control counseling, an STI test, STI treatment, a pregnancy test, or an annual gynecological or well-woman exam including a pap smear or HPV vaccination.

3 Compared to the final clinic universe (*n* = 38; eligibility criteria were sites in Iowa that served 100 or more family planning patients in 2018), the eligible clinics represented in the sample (*n* = 16) had higher proportions of sites serving 1000 + family planning patients annually (75% vs. 37%), Planned Parenthood clinics (50% vs. 21%), and Title X-funded sites (81% vs. 42%).

4 Given the policy under study going into effect in July 2017, the broadest time period that this retrospective survey item would have covered would have been between May 2017 and February 2018. As such, we consider almost all responses to this item to reflect on the post-policy context.

5 We strove to use language related to contraception that would be most familiar to respondents. Based on past surveys with family planning patients and after pilot testing our baseline instrument, we elected to use the phrase “birth control” in all of the surveys, with the following clarifying language prior to any questions including this phrase: Birth control refers to ALL the different methods for preventing pregnancy, including condoms, birth control pills, Depo-Provera (the shot), IUDs, non-prescription methods like withdrawal or “pulling out,” permanent methods like sterilization (tubes tied or vasectomy),” and other methods, even if you used them for reasons other than pregnancy prevention.

6 The analytic sample roughly aligns with the full baseline survey sample, those from the baseline who did not opt-in to participating in follow-up surveys, and those from the baseline who did opt in but did not complete any follow-up surveys on having received baseline care at a Title X site, income, race/ethnicity, and sexual identity. The analytic sample aligns with these three populations on age breakdowns 30–39 and 40 + , but includes higher proportions of young adults ages 20–29 (62% compared to 52–55%) and lower proportions of adolescents ages 15–19 (15% compared to 21–23%). Slightly higher proportions of the analytic sample reported having received baseline care at a Medicaid restricted site (82% compared to 68–73%) and at least some college or associate-level education (75% compared to 60–65%).

7 Note that the full sample received recent care at the first 6-month follow-up survey time point. We considered any respondent who did not directly report having received recent care at that 6-month follow-up to have received recent care because they had received care at the baseline facility where they enrolled in the study, which occurred approximately 6 months prior to completing the first 6-month follow-up survey.
